# Supplementary material for: Antisense transcription as a tool to tune gene expression
Source: Mol Syst Biol. 2016 Jan 13;12(1):854. doi: 10.15252/msb.20156540 (PMC4731013; doi:10.15252/msb.20156540)
Supplement: Supplementary file 1 — Appendix [file MSB-12-854-s001.pdf]

Appendix for

Antisense transcription as a tool to tune gene expression

Jennifer A.N. Brophy and Christopher A. Voigt

Contents

Appendix figures and tables

Appendix Figure S1: Reverse transcription reporter plasmids.

Appendix Figure S2: Growth curves of cells harboring antisense reporter plasmids.

Appendix Figure S3: Correlations between bin fluorescence and part strengths.

Appendix Figure S4: Sorted gates vs. terminator strength.

Appendix Figure S5: Plasmids to measure repression generated by asRNA produced in *trans.*

Appendix Figure S6: Model results when ε_F and ε_R = 0 – 1 at increments of 0.01.

Appendix Figure S7: Best fit values of ε as a function of P_F and P_R promoter strength.

Appendix Figure S8: Reverse transcription reporter plasmids to vary the distance between promoters *N*.

Appendix Figure S9: NOT gate plasmids used in this study.

Appendix Figure S10: Plasmids to measure promoter firing rates (RNAP/second)

Appendix Figure S11: REU standard plasmids used in this study.

Appendix Figure S12: Fluorescence histograms corresponding to the data in Figure 1c.

Appendix Figure S13: Fold-repression θ generated by additional antisense promoters in trans.

Appendix Table S1: Illumina sequencing results.

Appendix Table S2: Terminator/promoter pairs tested.

Appendix Table S3: Collision interference model parameters.

Appendix Table S4: Oligonucleotides used in this study.

Appendix Table S5: Promoter and Terminator ordering for Figure EV4

Appendix Figures:

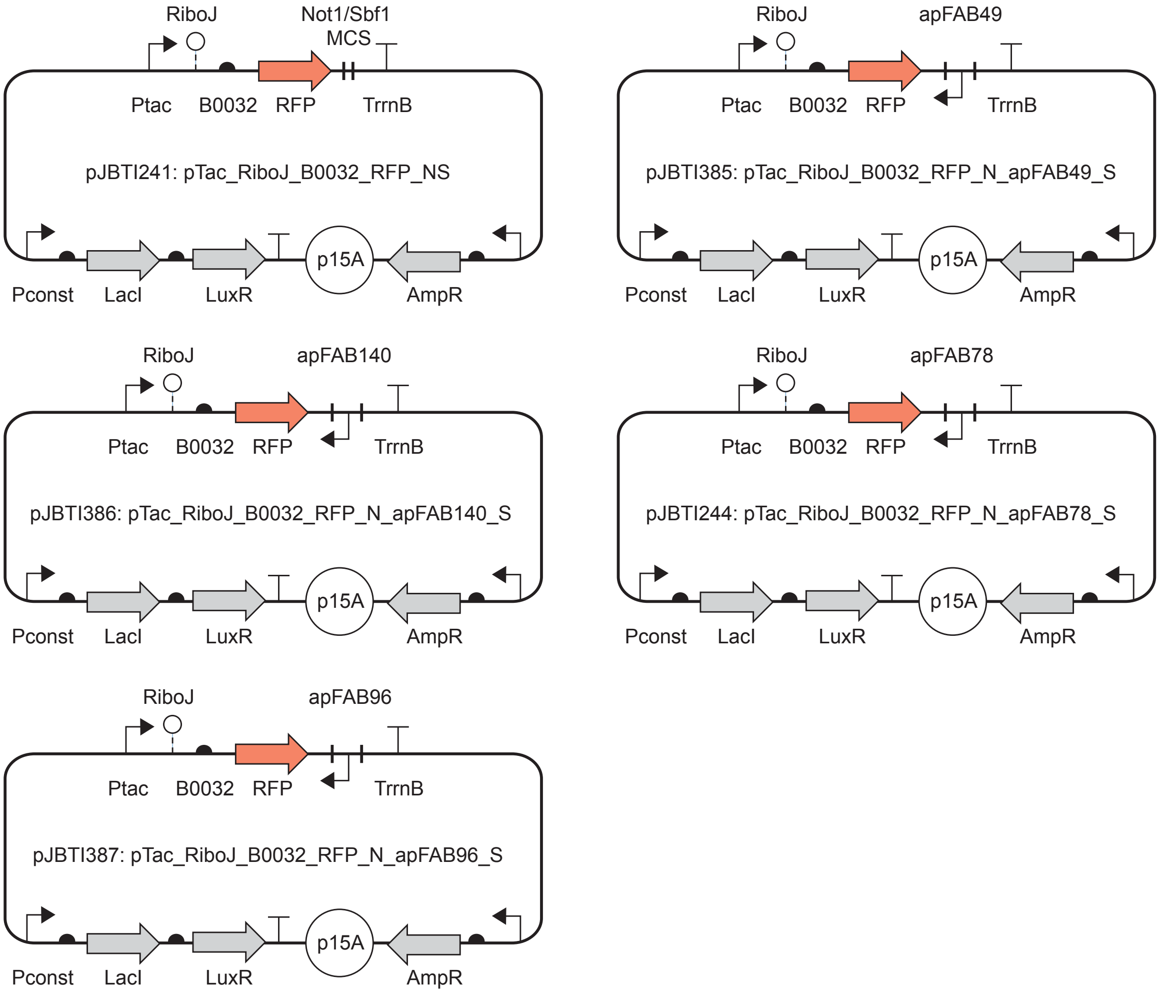

Appendix Figure S1: **Reverse transcription reporter plasmids.** pJBTI241 was used to measure P_{tac} driven expression of RFP. Not1/Sbf1 multiple cloning sites were used to digest pJBTI241 and insert promoters (apFAB49, apFAB140, apFAB78, apFAB96) at the 3'end of *rfp*, yielding plasmids pJBTI385, pJBTI386, pJBTI244, and pJBTI387. These plasmids were used to measure RFP expression with and without antisense promoters and quantify antisense transcription-mediated repression.

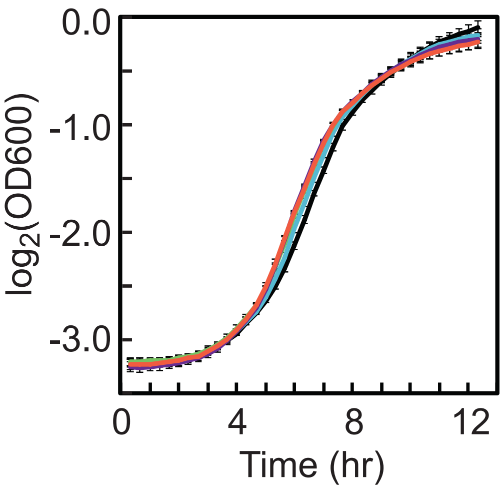

<u>Appendix Figure S2:</u> **Growth curves of cells harboring antisense reporter plasmids.** Constitutive antisense promoters do not cause growth defects. No antisense promoter (JBTI241; black), apFAB49 (JBTI385; blue), apFAB140 (JBTI386; green), apFAB78 (JBTI244; purple), apFAB96 (JBTI387; red). Error bars are the s.d. of three biological replicates.

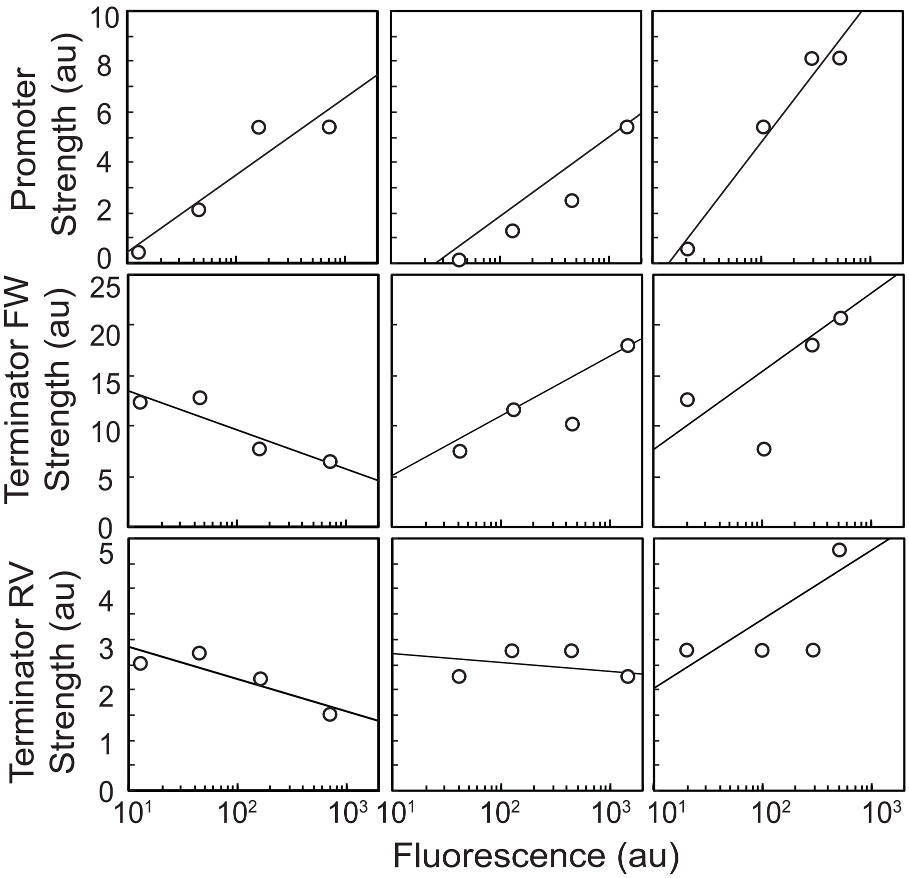

Appendix Figure S3: Correlations between bin fluorescence and part strengths. Median strengths of all composite parts sorted into bins in the PhlF (Left), SrpR (Center) and TarA (Right) libraries as a function of median bin fluorescence. Antisense promoter strengths correlate positively with bin fluorescence; PhlF R^2 = 0.87421, SrpR R^2 = 0.96061, TarA R^2 = 0.96112 (logarithmic regression). Forward and reverse terminator strengths do not show consistent trends across the libraries; PhlF FW R^2 = 0.84079, PhlF RV R^2 = 0.78599, SrpR FW R^2 = 0.8628, SrpR RV R^2 = 0.16902, TarA FW R^2 = 0.71405, TarA RV R^2 = 0.74852 (logarithmic regression).

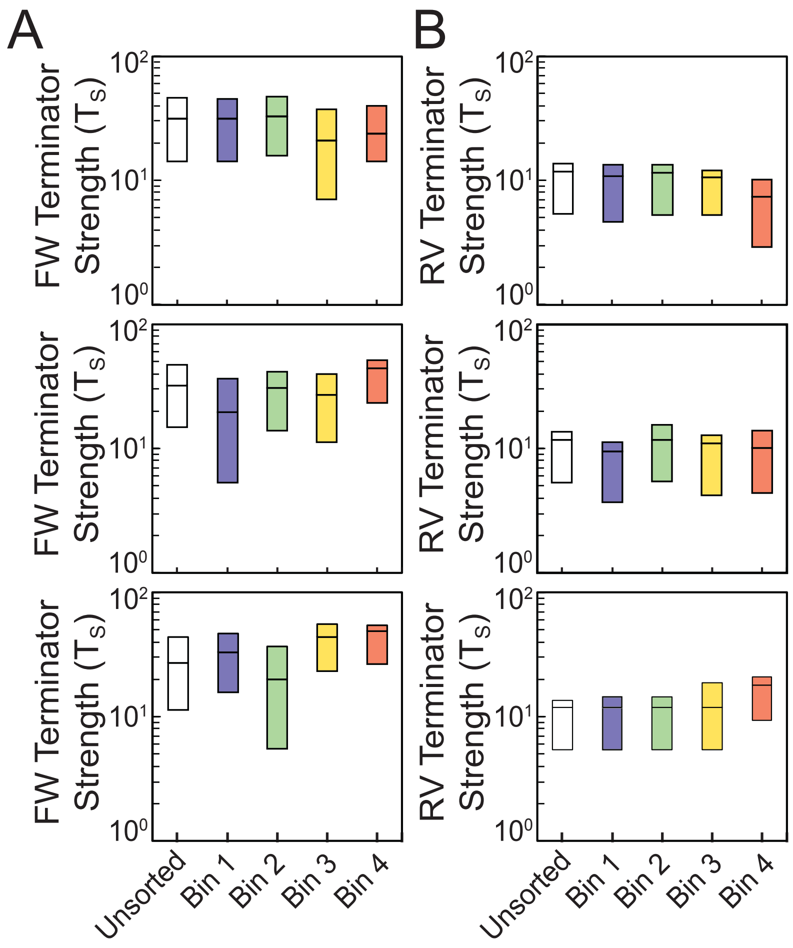

<u>Appendix Figure S4:</u> **Sorted gates vs. terminator strength.** (a) Forward and (b) reverse terminator strengths in the unsorted (white) and sorted (Bins 1p 4) libraries. PhlF (top), SrpR (middle), TarA (bottom). All terminator strengths were measured in a previous study (Chen et al, 2013). Box plots display the median part strength, with hinges indicating the first and third quartiles.

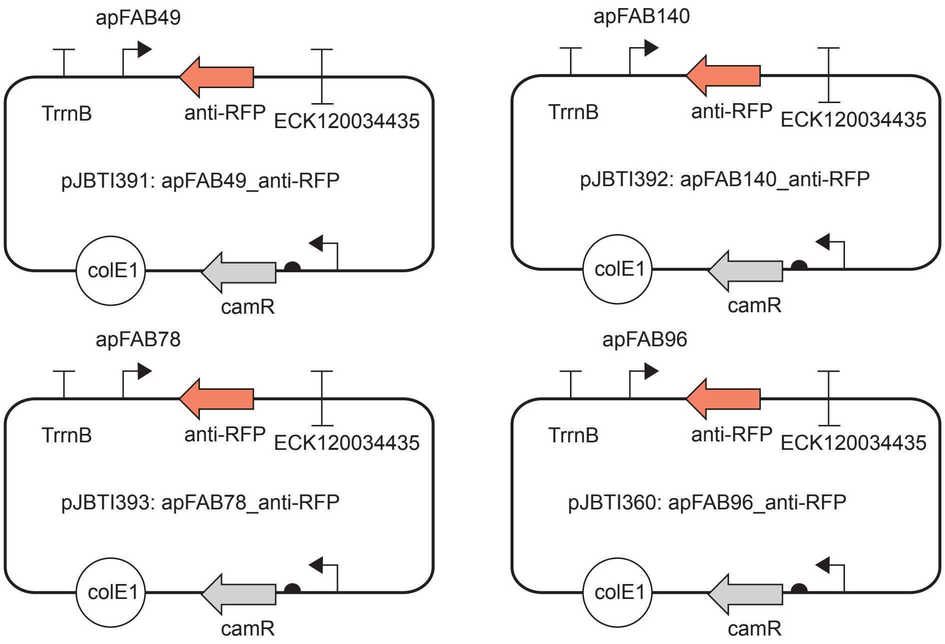

<u>**Appendix Figure S5:**</u> **Plasmids to measure repression generated by asRNA produced in *trans*.** These plasmids were co-transformed with pJBTI241 and RFP fluorescence was measured to test repression generated by asRNA in *trans.*

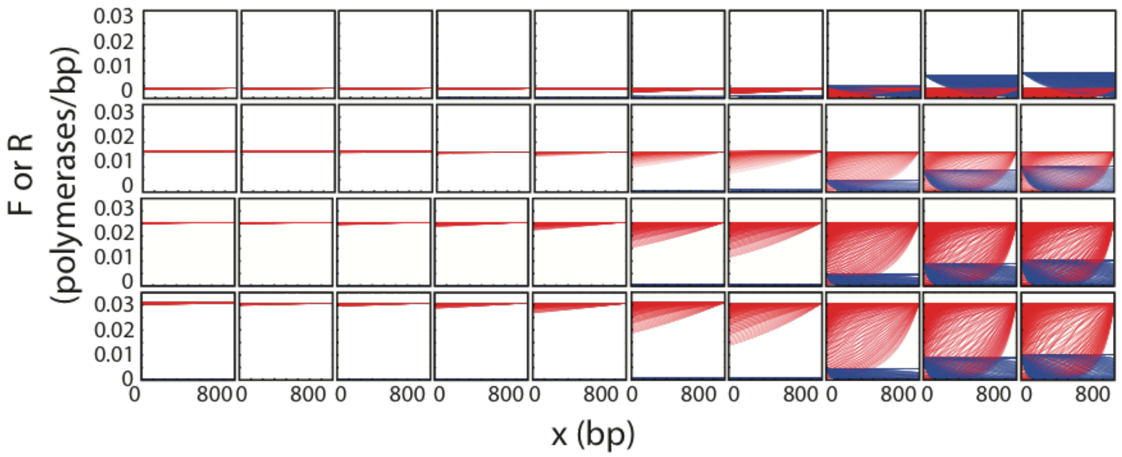

<u>Appendix Figure S6:</u> **Model results when ε_F and ε_R = 0 – 1 at increments of 0.01.** Graphs show density of polymerases fired from either the forward (F - blue) or interfering promoter (R - red) along the DNA. Forty different forward/interfering promoter combinations were simulated, which model P_F as pTac + ten IPTG concentrations (1, 5, 10, 20, 50, 70, 100, 200, 500, 1000 μM; across) and P_R as apFAB49 (top row), apFAB140 (second row), apFAB78 (third row), or apFAB96 (bottom row).

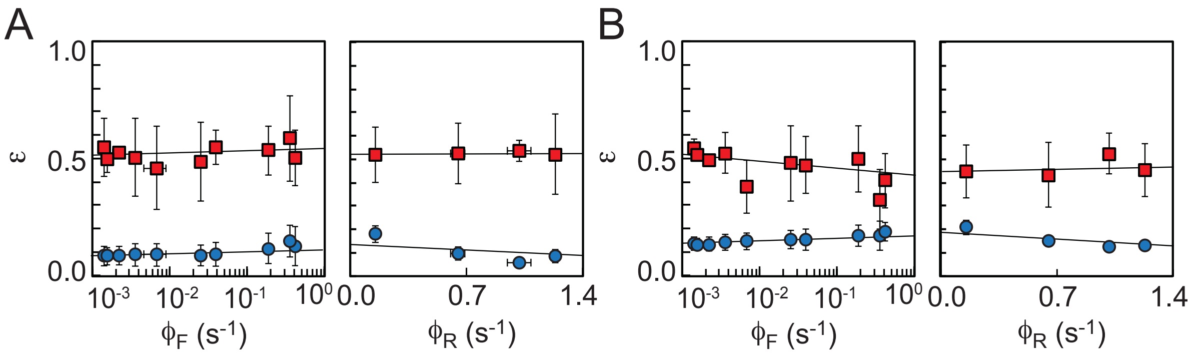

<u>**Appendix Figure S7:**</u> **Best fit values of ε as a function of forward P_F and antisense P_R promoter strength.** Relationships between forward promoter strengths, ϕ_F and ϕ_R, and the probabilities that polymerases fall off the DNA after collision, ε_F (blue circles) and ε_R (red squares). Graphs show the best ε_F and ε_R for each promoter combination from the ε parameter sweep and experimentally measured ϕ_F and ϕ_R. ε_F and ε_R were fit to experimental data of fold repression generated by transcriptional interference alone θ_{TI} (a) or by maximum fold repression (b). The highest scoring ε_Fs and $\varepsilon_R s$ for each ϕ_F and ϕ_R are averaged, y-error bars show the s.d. between these values. x-error bars show the s.d. of three replicates collected on different days.

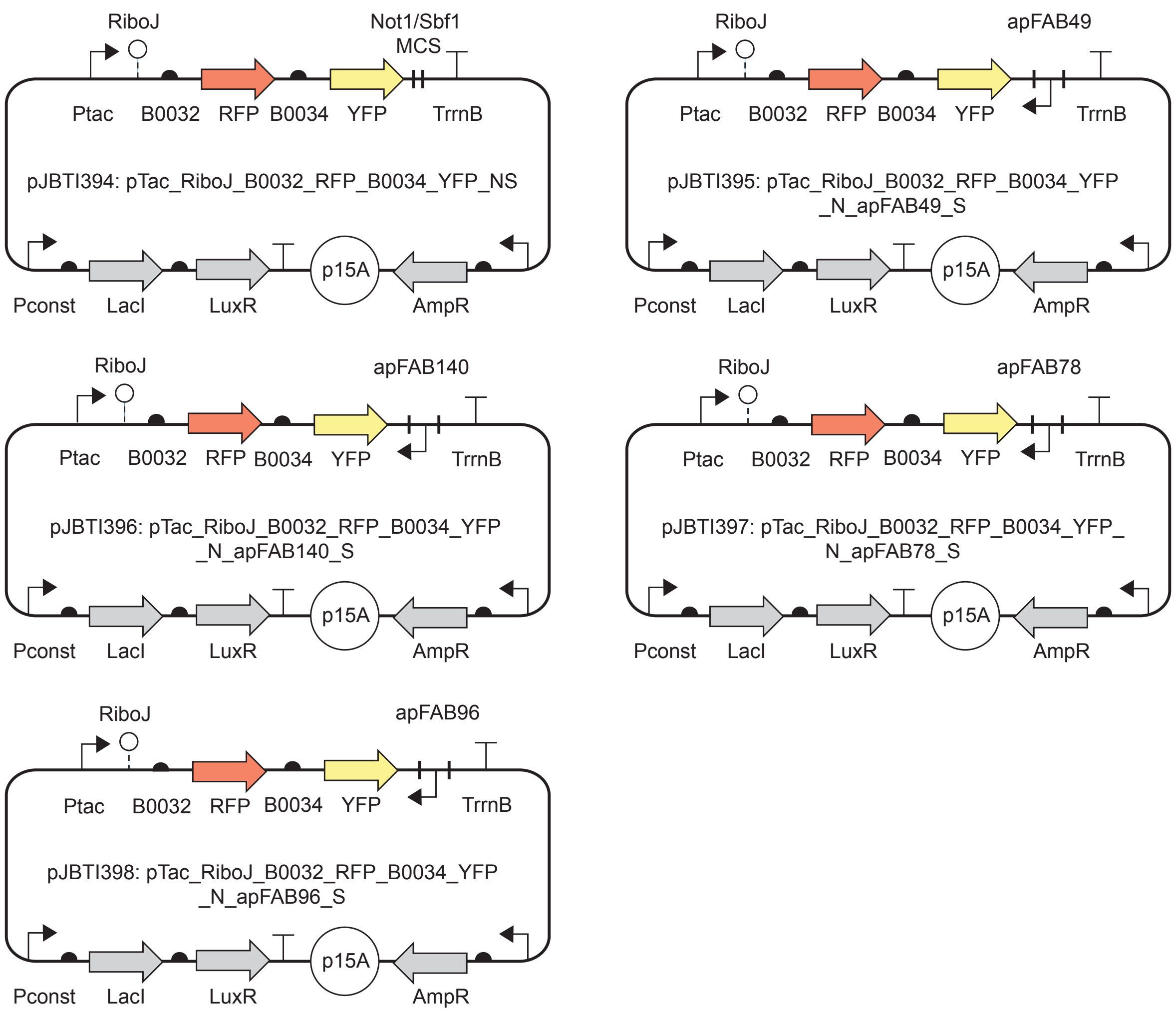

Appendix Figure S8: **Reverse transcription reporter plasmids to vary the distance between promoters *N*.** pJBTI394 was used to measure P_{tac} driven expression of RFP and YFP. Not1/Sbf1 multiple cloning sites were used to digest pJBTI394 and insert promoters (apFAB49, apFAB140, apFAB78, apFAB96) at the 3'end of *yfp*, yielding plasmids pJBTI395, pJBTI396, pJBTI397, and pJBTI398. These plasmids were used to measure RFP and YFP expression with and without antisense promoters and quantify antisense transcription-mediated repression.

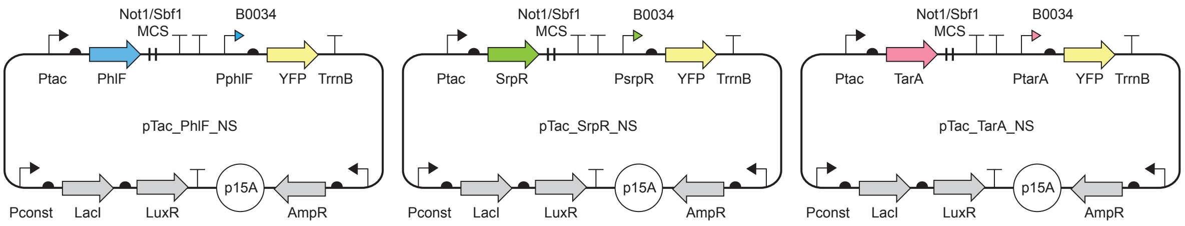

Appendix Figure S9: **NOT gate plasmids used in this study.** All NOT gate plasmids have Not1/Sbf1 multiple cloning sites used to insert terminator/antisense promoter oligonucleotide library.

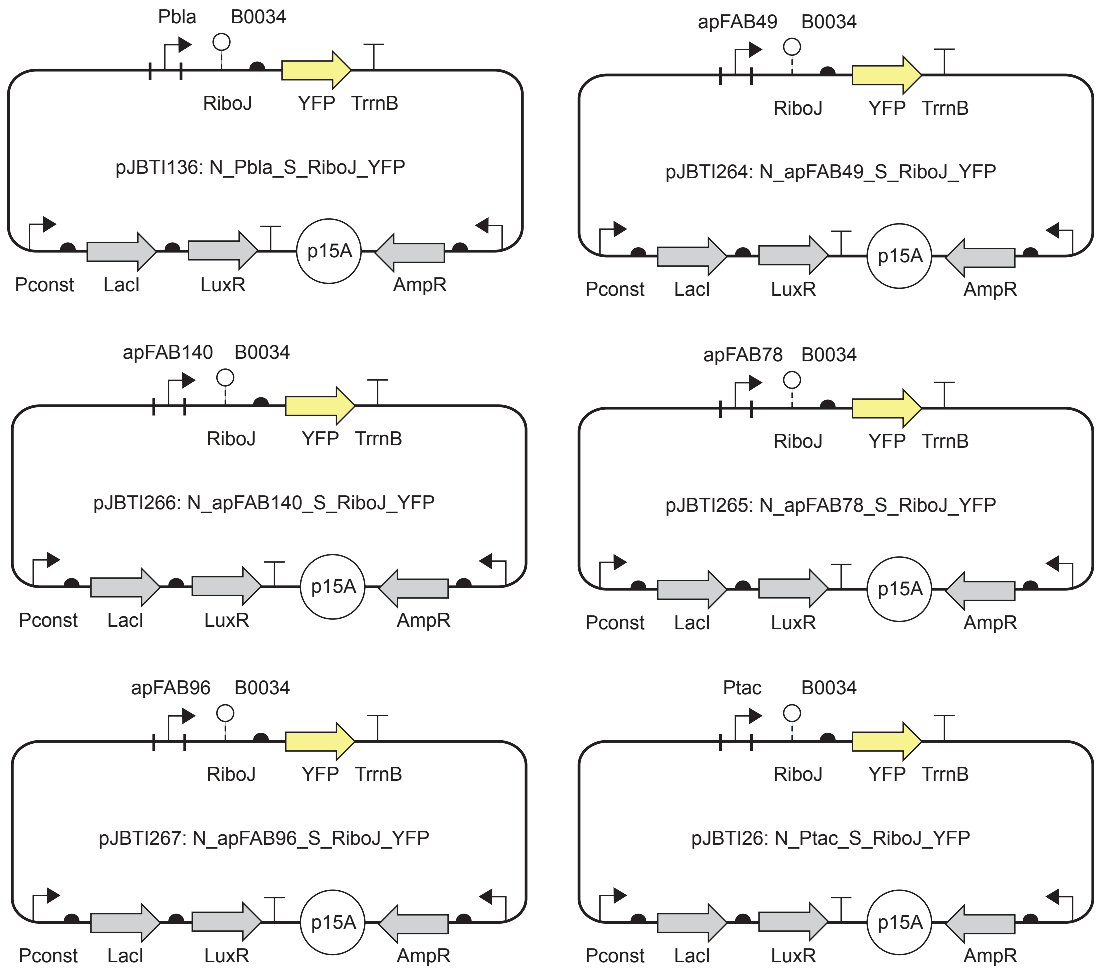

Appendix Figure S10: **Plasmids to measure promoter firing rates (RNAP/second)** Promoter Pbla was used to define a polymerase firing rate of 0.031 s^{-1}. Not1/Sbf1 multiple cloning sites were used to digest the reference plasmid and insert other promoters (apFAB49, apFAB140, apFAB78, apFAB96, Ptac) for promoter strength measurement.

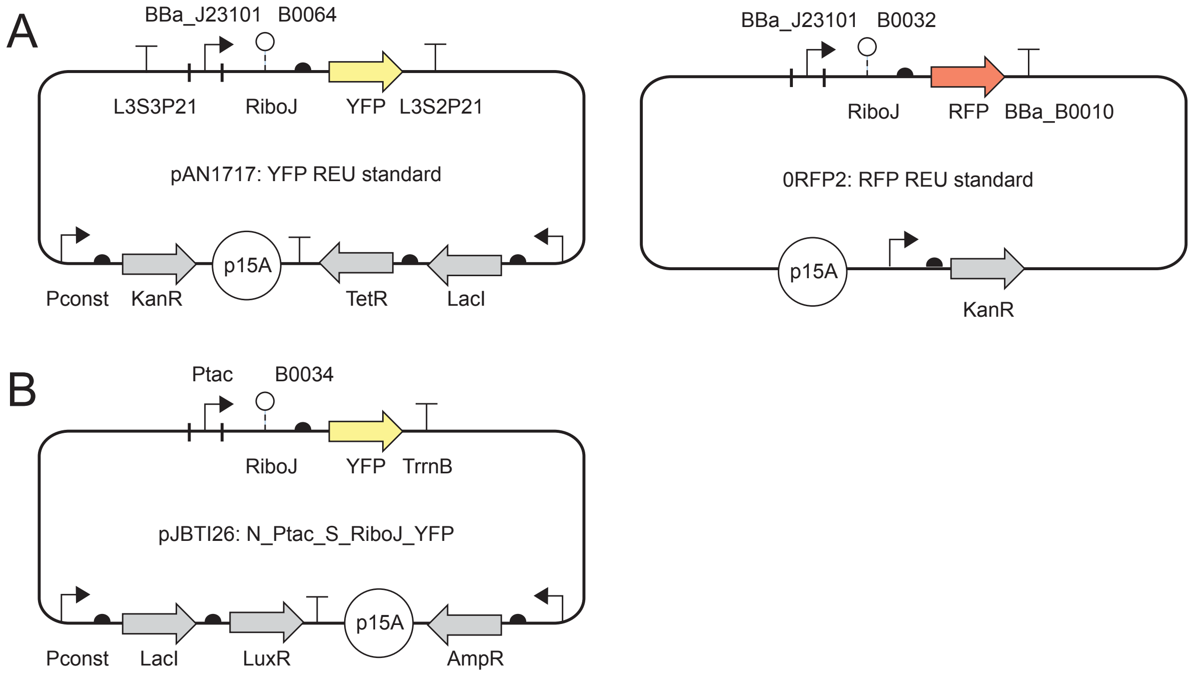

__Appendix Figure S11:__ **REU standard plasmids used in this study. (A)** Plasmids used to convert YFP (pAN1717) and RFP (0RFP2) fluorescence measurements into Relative Expression Units (REUs) (Supplementary Methods). **(B)** Plasmid used to measure input (P_{tac}) promoter activity for NOT gate response functions.

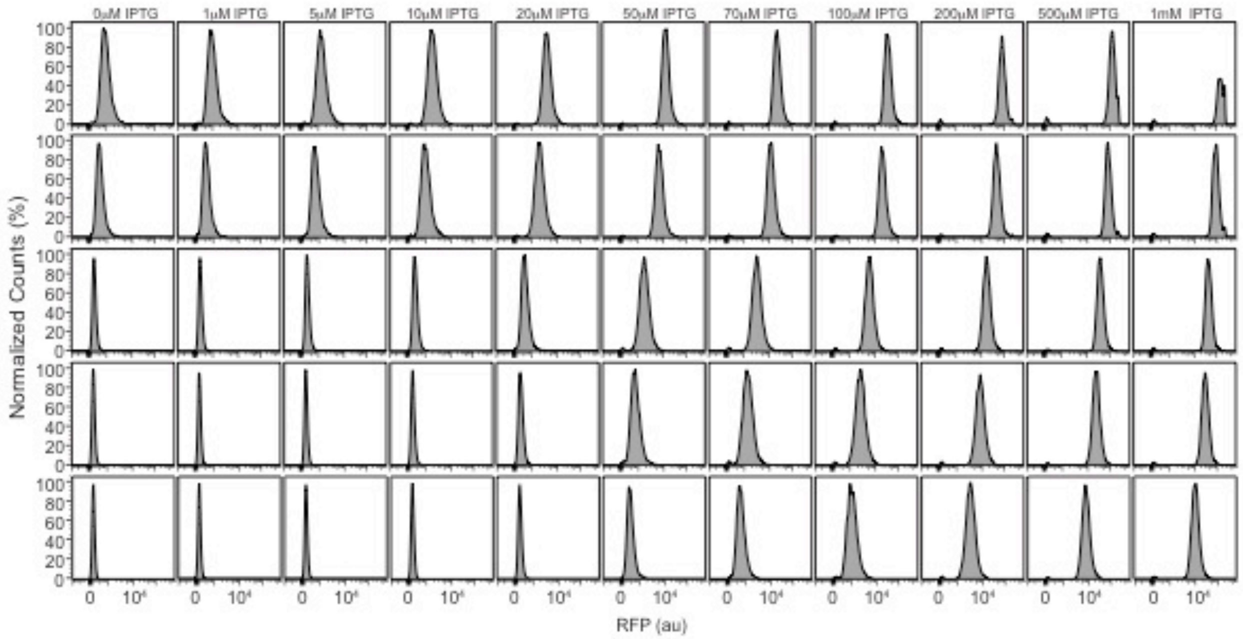

<u>Appendix Figure S12:</u> Fluorescence histograms corresponding to the data in Figure 1c. Antisense promoter: none (JBTI241 - top row), apFAB49 (JBTI385 - second row), apFAB140 (JBTI386 - third row), apFAB78 (JBTI244 - fourth row), apFAB96 (JBTI387 - bottom row).

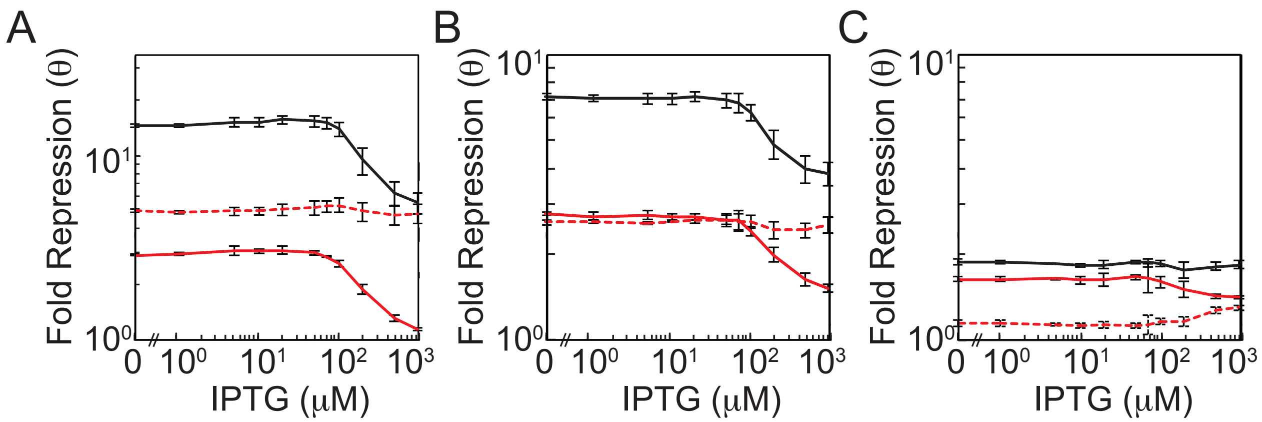

Appendix Figure S13: **Fold-repression θ generated by additional antisense promoters in trans.** Total fold repression generated by antisense promoters (a - apFAB78, b – apFAB140, c – apFAB49) is shown as a function of forward promoter activity using the characterization system in Figure 1A (black line). This is compared to the repression observed when the same promoters are used to drive the transcription of asRNA in *trans* from a separate plasmid (dashed red line) (Figure EV4). The repression due to transcriptional interference θ_{TI} (solid red line) is inferred from the total and *trans* asRNA repression data.

Appendix Tables:

	Total	Unpaired		Paired		Not1/Sbf1 (-)		Not1/Sbf1 (+)		Perfect	
		Reads	%	Reads	%	Reads	%	Reads	%	Reads	%
**PhlF**											
**Unsorted**	2,187,634	258,535	11.82%	1,929,099	88.18%	79,033	4.10%	1,850,066	84.57%	607,711	32.85%
**BIN1**	2,004,210	239,025	11.93%	1,765,185	88.07%	72,371	4.10%	1,692,814	84.46%	548,928	32.43%
**BIN2**	167,601	12,946	7.72%	154,655	92.28%	5,204	3.36%	149,451	89.17%	46,927	31.40%
**BIN3**	125,143	13,686	10.94%	111,457	89.06%	4,628	4.15%	106,829	85.37%	34,268	32.08%
**BIN4**	106,160	10,798	10.17%	95,362	89.83%	3,369	3.53%	91,993	86.66%	35,642	38.74%
**SrpR**											
**Unsorted**	1,663,731	198,112	11.91%	1,465,619	88.09%	75,217	5.13%	1,390,402	83.57%	431,538	31.04%
**BIN1**	1,872,389	225333	12.03%	1647056	87.97%	63961	3.88%	1583095	84.55%	497,718	31.44%
**BIN2**	122,744	12,629	10.29%	110,115	89.71%	5,097	4.63%	105,018	85.56%	39,937	38.03%
**BIN3**	153,442	13,987	9.12%	139,455	90.88%	4,774	3.42%	134,681	87.77%	37,026	27.49%
**BIN4**	163,519	12,563	7.68%	150,956	92.32%	4,770	3.16%	146,186	89.40%	49,411	33.80%
**TarA**											
**Unsorted**	2,171,123	198,140	9.13%	1,972,983	90.87%	76,631	3.88%	1,896,352	87.34%	622,713	32.84%
**BIN1**	1,910,985	184,335	9.65%	1,726,650	90.35%	77,528	4.49%	1,649,122	86.30%	517,513	31.38%
**BIN2**	180,667	18,879	10.45%	161,788	89.55%	6,060	3.75%	155,728	86.20%	57,696	37.05%
**BIN3**	157,354	23,898	15.19%	133,456	84.81%	6,502	4.87%	126,954	80.68%	51,839	40.83%
**BIN4**	143,926	17,630	12.25%	126,296	87.75%	4,831	3.83%	121,465	84.39%	47,742	39.31%

Appendix Table S1: **Illumina sequencing results.**

Terminator	Promoter	Maximum repression ± s.d.
ECK120035132	apFAB71	61.86 ± 33.25
ECK120010831	apFAB71	36.60 ± 12.32
ECK120021270	apFAB67	23.08 ± 1.50
ECK120021270	apFAB61	17.94 ± 2.29
ECK120010793	apFAB61	12.84 ± 1.54
ECK120034435	apFAB341	7.05 ± 0.63
ECK120030221	Bba_J23119	7.16 ± 0.75
ECK120010815	apFAB345	1.49 ± 0.02
ECK120010831	apFAB345	1.51 ± 0.18
ECK120035132	Bba_J23102	1.44 ± 0.06
ECK120010793	apFAB69	1.26 ± 0.03
ECK120034435	apFAB259	1.23 ± 0.17
ECK120030221	apFAB259	0.98 ± 0.04
ECK120010836-R	Bba_J23109	0.87 ± 0.03

Appendix Table S2: **Terminator/promoter pairs tested in Figure 3c.** Maximum repression is the average of three replicates collected on different days.

Parameter	Meaning	Values	References
N	Distance between transcription start points (bp)	841	
v	Speed of transcription (bp/s)	40	37, 72
ϕ_F	Rate of RNAP production from P_F (s-1)	pBla: 0.031	62
		pTac [1]: 0.001439	this study
		pTac [5]: 0.001548	this study
		pTac [10]: 0.002232	this study
		pTac [20]: 0.003643	this study
		pTac [50]: 0.006778	this study
		pTac [70]: 0.025262	this study
		pTac [100]: 0.03976	this study
		pTac [200]: 0.18756	this study
		pTac [500]: 0.35764	this study
		pTac [1000]: 0.41141	this study
ϕ_R	Rate of RNAP production from P_R (s-1)	apFAB49: 0.15345	this study
		apFAB140: 0.64814	this study
		apFAB78: 1.01410	this study
		apFAB96: 1.23185	this study
ε_F	Ejection factor for RNAP fired from P_F	0.104 $\pm$ 0.031	fitted
ε_R	Ejection factor for RNAP fired from P_R	0.494 $\pm$ 0.027	fitted

Appendix Table S3: **Model parameters**

**Amplify oligonucleotides from chip synthesized pool:**		
oj1299	FW	ATATAGATGCCGTCCTAGCGGCG
oj1300	RV	TGGGCACAGGAAAGATACTTCCTG
**Add deep sequencing barcodes:**		
oj1302	FW amplification primer:	AATGATACGGCGACCACCGAGATCTACACgtgacatTAACTAGGGCGCGGCCGC
oj1334	RV PhlF BIN1 amplification primer	CAAGCAGAAGACGGCATACGAGATGTGTGTgcttctcgccaTGGGGGTATGGCCTGCAGG
oj1335	RV PhlF BIN2 amplification primer	CAAGCAGAAGACGGCATACGAGATAGGTGTgcttctcgccaTGGGGGTATGGCCTGCAGG
oj1336	RV PhlF BIN3 amplification primer	CAAGCAGAAGACGGCATACGAGATTCGTGTgcttctcgccaTGGGGGTATGGCCTGCAGG
oj1337	RV PhlF BIN4 amplification primer	CAAGCAGAAGACGGCATACGAGATCAGTGTgcttctcgccaTGGGGGTATGGCCTGCAGG
oj1338	RV PhlF Unsorted amplification primer	CAAGCAGAAGACGGCATACGAGATGTAGGTgcttctcgccaTGGGGGTATGGCCTGCAGG
oj1339	RV SrpR BIN1 amplification primer	CAAGCAGAAGACGGCATACGAGATGTGTTCgcttctcgccaTGGGGGTATGGCCTGCAGG
oj1340	RV SrpR BIN2 amplification primer	CAAGCAGAAGACGGCATACGAGATCATGACgcttctcgccaTGGGGGTATGGCCTGCAGG
oj1341	RV SrpR BIN3 amplification primer	CAAGCAGAAGACGGCATACGAGATGTTCTCgcttctcgccaTGGGGGTATGGCCTGCAGG
oj1342	RV SrpR BIN4 amplification primer	CAAGCAGAAGACGGCATACGAGATAGTCTCgcttctcgccaTGGGGGTATGGCCTGCAGG
oj1343	RV SrpR Unsorted amplification primer	CAAGCAGAAGACGGCATACGAGATTCTCTCgcttctcgccaTGGGGGTATGGCCTGCAGG
oj1344	RV TarA BIN1 amplification primer	CAAGCAGAAGACGGCATACGAGATTGCAGAgcttctcgccaTGGGGGTATGGCCTGCAGG
oj1345	RV TarA BIN2 amplification primer	CAAGCAGAAGACGGCATACGAGATGTATCAgcttctcgccaTGGGGGTATGGCCTGCAGG
oj1346	RV TarA BIN3 amplification primer	CAAGCAGAAGACGGCATACGAGATTCTCCAgcttctcgccaTGGGGGTATGGCCTGCAGG
oj1347	RV TarA BIN4 amplification primer	CAAGCAGAAGACGGCATACGAGATCATCCAgcttctcgccaTGGGGGTATGGCCTGCAGG
oj1348	RV TarA Unsorted amplification primer	CAAGCAGAAGACGGCATACGAGATGTCACAgcttctcgccaTGGGGGTATGGCCTGCAGG
**Sequencing primers:**		
oj1301	FW seq primer:	gtgacatTAACTAGGGCGCGGCCGC
oj1303	RV seq primer:	gcttctcgccaTGGGGGTATGGCCTGCAGG
oj1356	Barcode read:	CCTGCAGGCCATACCCCCAtggcgagaagc

Appendix Table S4: **Oligonucleotides used in this study**

Terminators		Promoters			
Number	Name	Number	Name	Number	Name
1	ECK120010843-R	1	apFAB46	56	apFAB33
2	ECK120010802	2	apFAB101	57	apFAB339
3	ECK120010813-R	3	apFAB96	58	apFAB321
4	ECK120010780	4	apFAB47	59	apFAB115
5	ECK120029530-R	5	apFAB42	60	apFAB125
6	ECK120015446-R	6	apFAB95	61	apFAB82
7	BBa_B0051	7	apFAB36	62	j23102
8	ECK120030672	8	apFAB68	63	apFAB346
9	ECK120035132	9	apFAB31	64	apFAB317
10	ECK120015439-R	10	apFAB93	65	apFAB63
11	ECK120026315-R	11	apFAB54	66	apFAB64
12	ECK120010831	12	apFAB92	67	apFAB322
13	ECK120010841-R	13	apFAB52	68	apFAB150
14	ECK120020528	14	apFAB81	69	apFAB69
15	ECK120010796	15	apFAB71	70	j23101
16	ECK120010874	16	apFAB67	71	apFAB104
17	ECK120020622	17	apFAB70	72	apFAB89
18	ECK120010857	18	apFAB79	73	apFAB306
19	ECK120030802	19	apFAB100	74	apFAB338
20	ECK120021270	20	apFAB45	75	apFAB58
21	ECK120011170-R	21	apFAB61	76	apFAB302
22	ECK120010852	22	apFAB341	77	apFAB73
23	ECK120020525	23	apFAB80	78	j23118
24	ECK120010832-R	24	apFAB29	79	apFAB342
25	ECK120010833	25	apFAB32	80	apFAB87
26	ECK120010812-R	26	apFAB40	81	j23106
27	ECK120010836-R	27	apFAB30	82	apFAB49
28	ECK120010871	28	apFAB56	83	apFAB38
29	ECK120010806	29	j23119	84	apFAB130
30	ECK120030221	30	apFAB50	85	apFAB98
31	ECK120010825	31	apFAB85	86	apFAB312
32	ECK120015457-R	32	apFAB53	87	apFAB129
33	ECK120010840	33	apFAB318	88	apFAB311
34	ECK120010781	34	apFAB44	89	j23107
35	ECK120010864	35	apFAB76	90	j23105
36	ECK120010793	36	apFAB65	91	apFAB277
37	ECK120010856	37	apFAB140	92	apFAB300
38	ECK120010867	38	apFAB66	93	j23110
39	ECK120010815	39	apFAB75	94	j23116
40	ECK120010863	40	apFAB345	95	j23114
41	ECK120010782	41	apFAB103	96	j23108
42	ECK120016882	42	apFAB347	97	apFAB259
43	ECK120023928	43	apFAB39	98	j23115
44	tonB/P14	44	apFAB94	99	j23109
45	ECK120035133	45	apFAB323	100	apFAB139
46	ECK120010855	46	apFAB97	101	j23113
47	ECK120017009	47	apFAB78	102	j23112
48	ECK120015170	48	apFAB57	103	apFAB134
49	ECK120026481-R	49	apFAB72	104	j23103
50	BBa_B0062	50	apFAB62	105	apFAB114
51	ECK120010858-R	51	apFAB48	106	j23117
52	ECK120034435	52	apFAB55	107	apFAB109
		53	j23100	108	apFAB149
		54	apFAB77	109	apFAB124
		55	j23104		

Appendix Table S5: **Promoter and Terminator ordering for Figure EV4**
