# Supplementary material for: Antisense transcription as a tool to tune gene expression
Source: Mol Syst Biol. 2016 Jan 13;12(1):854. doi: 10.15252/msb.20156540 (PMC4731013; doi:10.15252/msb.20156540)
Supplement: Supplementary file 2 — Expanded View Figures PDF [file MSB-12-854-s002.pdf]

Expanded View Figures

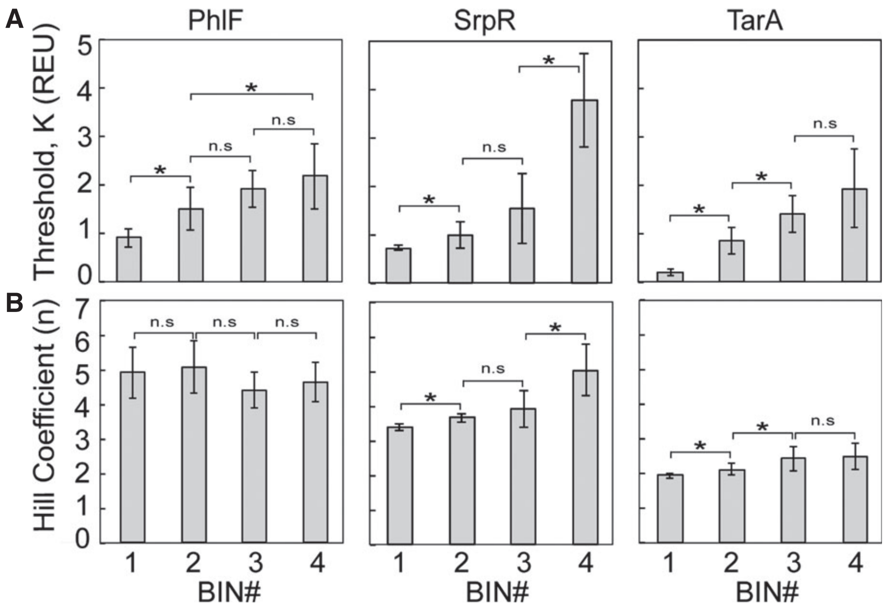

Figure EV1. Thresholds and Hill coefficients of sorted NOT gate constructs.

A Average thresholds *K* for NOT gates sorted into each bin of the PhlF (left), SrpR (middle), and TarA (right) libraries.

B Hill coefficients n for the same NOT gates.

Data information: Thresholds and Hill coefficients were calculated by fitting NOT gate response functions to the repressor Hill equation (equation 2). Error bars are the standard deviation between single measurements of the thresholds or Hill coefficients of the eight NOT gates characterized from each bin. Brackets indicate two-sample Student's *t*-tests with **P*-values < 0.05.

© 2016 The Authors

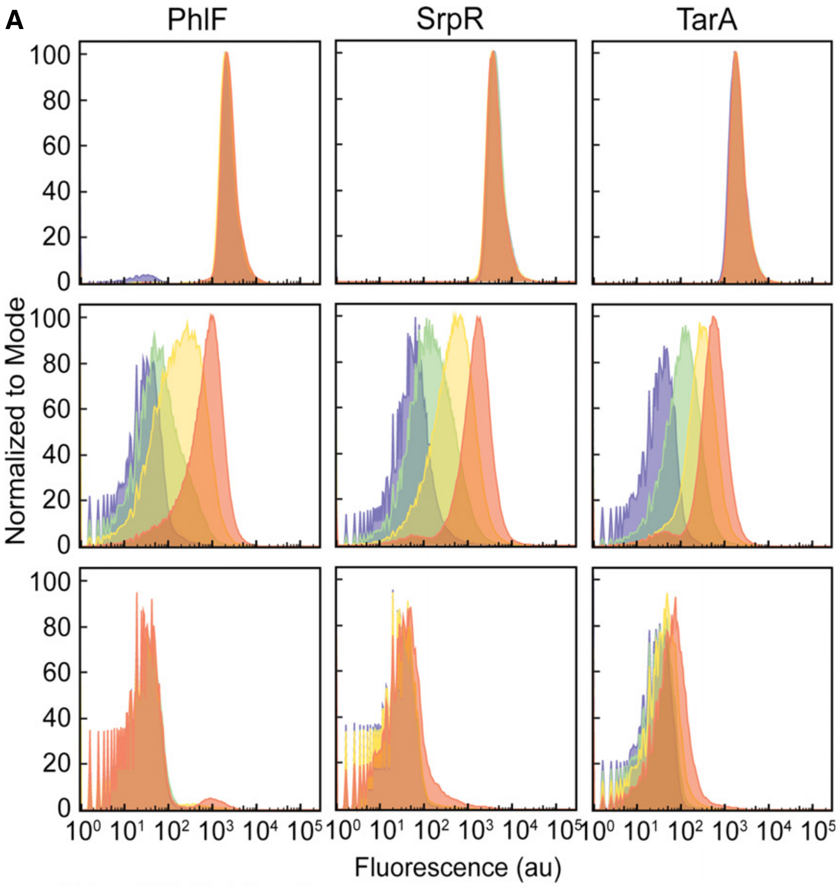

B Tukey HSD Test Results

Library	Bin1	Bin2	Bin3	Bin4
PhlF 100µM				
Mean (au)	66.53	112.39	385.05	846.92
s.d. (au)	78.77	163.60	959.71	620.12
P-value (Bin1–Bin2)	0.689			
P-value (Bin1–Bin3)	0.001			
P-value (Bin1–Bin4)	0.001			
P-value (Bin2–Bin3)		0.011		
P-value (Bin2–Bin4)		0.001		
P-value (Bin3–Bin4)			0.001	
SrpR 100µM				
Mean (au)	58.22	228.07	959.07	1,659.43
s.d. (au)	71.74	255.12	3805.81	1,284.53
P-value (Bin1–Bin2)	0.900			
P-value (Bin1–Bin3)	0.009			
P-value (Bin1–Bin4)	0.001			
P-value (Bin2–Bin3)		0.052		
P-value (Bin2–Bin4)		0.001		
P-value (Bin3–Bin4)			0.067	
TarA 100µM				
Mean (au)	15.77	141.09	523.55	587.32
s.d. (au)	30.72	139.58	1,277.83	601.34
P-value (Bin1–Bin2)	0.584			
P-value (Bin1–Bin3)	0.001			
P-value (Bin1–Bin4)	0.001			
P-value (Bin2–Bin3)		0.001		
P-value (Bin2–Bin4)		0.001		
P-value (Bin3–Bin4)			0.900	
TarA 1mM				
Mean (au)	6.46	16.63	44.81	86.41
s.d. (au)	28.78	43.54	172.14	87.92
P-value (Bin1–Bin2)	0.882			
P-value (Bin1–Bin3)	0.036			
P-value (Bin1–Bin4)	0.001			
P-value (Bin2–Bin3)		0.194		
P-value (Bin2–Bin4)		0.002		
P-value (Bin3–Bin4)			0.343	

Figure EV2. Fluorescence histograms of sorted bins.

A The fluorescence histogram of cells from the PhlF (left), SrpR (middle), and TarA (right) libraries. Fluorescence was measured using flow cytometry in ON (0 µM IPTG, top), threshold (100 µM IPTG, middle), and OFF (1,000 µM IPTG, bottom) conditions. Histograms are colored by bin: 1, purple; 2, green; 3, yellow; 4, orange. One hundred randomly selected individual cells from each data set were used as input to one-way ANOVA tests. There were no statistically significant differences between group means for the PhlF ON ($F(3, 396) = 1.06$, $P = 36.67$) and OFF ($F(3, 396) = 1.54$, $P = 20.48$) conditions, the SrpR ON ($F(3, 396) = 1.22$, $P = 30.04$) and OFF ($F(3, 396) = 1.41$, $P = 23.87$) conditions, or the TarA ON ($F(3, 396) = 1.25$, $P = 29.19$) condition. There are significant differences between group means for the threshold conditions: PhlF ($F(3, 396) = 34.67$, $P = 5.42E-18$), SrpR ($F(3, 396) = 13.3$, $P = 2.65E-8$), TarA ($F(3, 396) = 15.71$, $P = 1.13E-9$) and the TarA OFF condition ($F(3, 396) = 7.85$, $P = 0.0042$).

B *Post hoc* comparisons using the Tukey HSD test for the PhlF threshold, SrpR threshold, TarA threshold, and TarA OFF conditions. Mean fluorescence values of the groups are shown in arbitrary units (au). Brackets indicate Tukey HSD comparisons with *P*-values listed.

 © 2016 The Authors

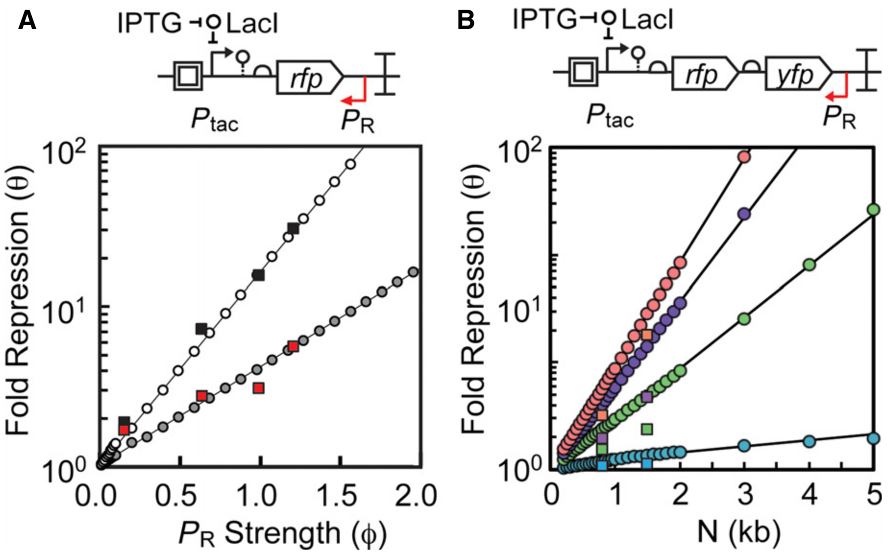

Figure EV3. Model predicts exponential increases in repression.

The collision interference model was used to predict fold repression with wide range of antisense promoter strength P_R values and different distances between promoters.

A Top: schematic showing the antisense transcription reporter system. Bottom: model simulations where ϕ_R is varied from 0 to 2 at increments of either 0.01 or 0.1 (1/s). For these simulations, ε_F was set to the median value fit by θ_{TI} (ε_F = 0.07, white circles) or θ (ε_F = 0.14, gray circles) and ε_R was held constant at 0.515. Experimental results for θ (black squares) and θ_{TI} (red squares) are also shown as a function of antisense promoter strength. Data from Fig 4A and Appendix Fig S13.

B Top: Schematic showing the antisense transcription reporter system with greater distance between P_F and P_R. A second fluorescent protein *yfp* was added between the 3′-end of *rfp* and P_R to increase the distance between promoters from 841 to 1,500 bp. Plasmid maps in Appendix Fig S8. Bottom: model simulations where N is varied from 200 to 5,000 bp in increments of 50, 100, or 1,000 bp. P_R was simulated as apFAB49, apFAB140, apFAB78, and apFAB96 by setting ϕ_R to values of 0.15345 RNAP/s (blue circles), 0.64814 RNAP/s (green circles), 1.014108 RNAP/s (purple circles), and 1.231852 RNAP/s (red circles), respectively. For all simulations in this figure, ϕ_F was held constant at 0.03976 (to simulate P_{tac} + 100 μM IPTG) and ε_F and ε_R were set at the median optimum values: 0.07 and 0.515, respectively. Experimental results show RFP and YFP repression with four different antisense promoters: apFAB49 (blue squares), apFAB140 (green squares), apFAB78 (purple squares), and apFAB96 (red squares).

Data information: Repression is measured when P_{tac} is induced with 100 μM IPTG.

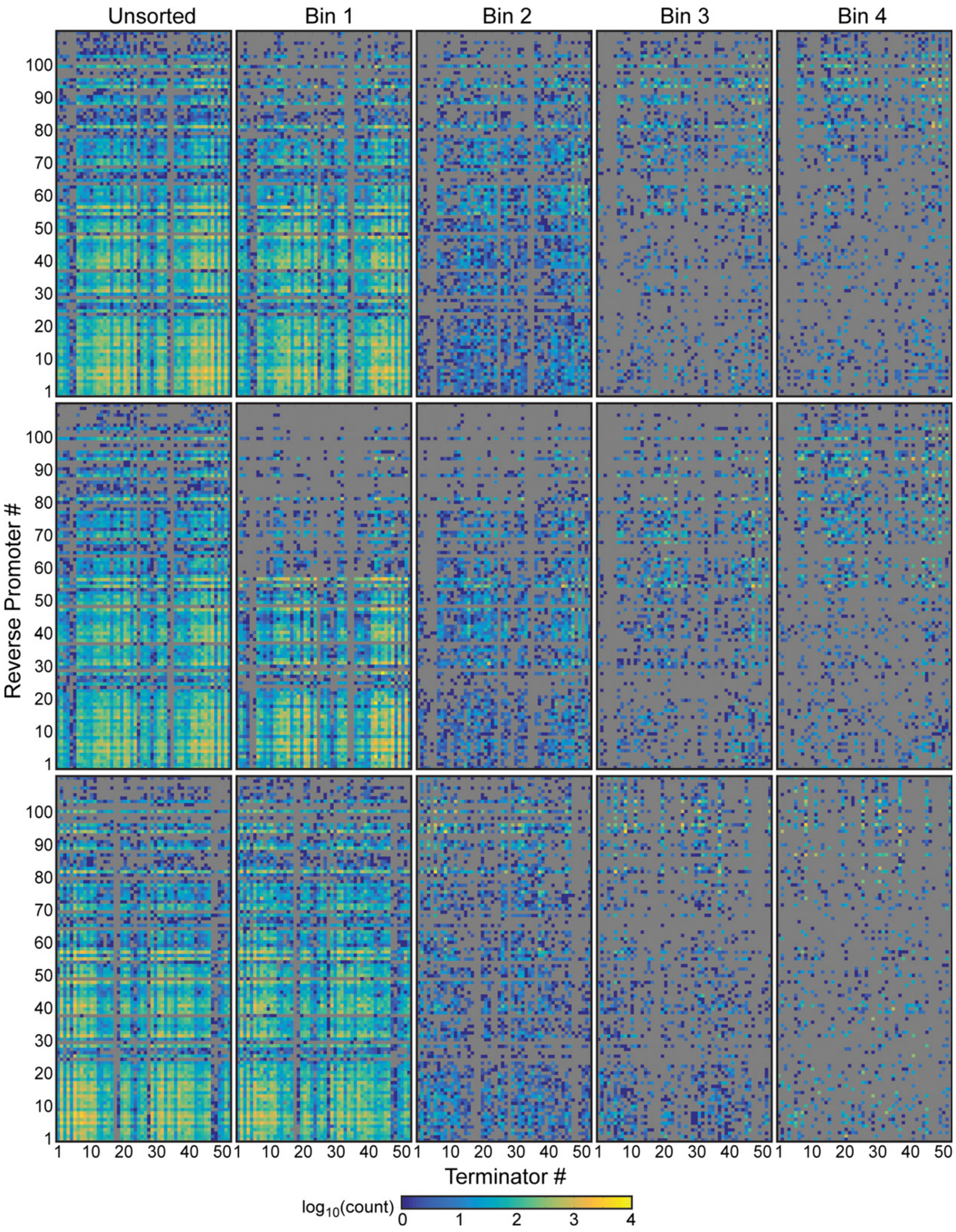

Figure EV4. Composite part frequencies in each library by bin.
Sequencing read counts for each composite part in the PhlF (top), SrpR (middle), and TarA (bottom) libraries. Only reads that are perfect matches to the designed sequences are shown. Promoter and terminator ordering is the same for all heatmaps. Promoters are ordered by strength from 1 (weakest) to 109 (strongest). Reference spreadsheet is available as Appendix Table S5.

 © 2016 The Authors
