# Supplementary material for: Antisense transcription as a tool to tune gene expression
Source: Mol Syst Biol. 2016 Jan 13;12(1):854. doi: 10.15252/msb.20156540 (PMC4731013; doi:10.15252/msb.20156540)
Supplement: Supplementary file 3 — Code EV1 [file MSB-12-854-s003.zip › Computer_code_EV1/readme.docx]

**illuminaSeqAnalysis_2.m**

A script to combine paired end reads from an illumine MiSeq run by homology between reads. Restriction enzyme recognition sequences that flank designed oligonucleotides are identified and flanking sequences are removed. Resulting oligonucleotides are compared to designed sequences to identify constructs in each sequencing sample (Brophy et al. 2015).

**Usage** (MATLAB script; requires MATLAB):

illuminaSeqAnalysis_2(A,B)

**Required input files:**

A <seqfile> Input forward reads sequence file name. Sequence input in FASTA format.

B <seqfile> Input reverse reads sequence file name. Sequence input in FASTA format.

‘TI_oligo_sequences’ <referencefile> Reference file of designed oligonucleotides that served as input to the library along with constituent part names, sequences and part strengths. XLSX format.

**Output file:**

Tab delimited text file, of all sequences in sample that are a perfect match to the designed library. Best viewed in Excel or equivalent. Column headers as follows:

Counts Number of times the oligonucleotide sequence appears in the sample.

Terminator name Name of the terminator in this construct.

Terminator fw strength Forward strength of this terminator. Previously measured by Chen et. al, 2013.

Terminator rv strength Antisense strength of this terminator. Previously measured by Chen et. al, 2013.

Promoter name Name of the promoter in this construct.

Promoter strength Strength of this promoter. Previously measured by Kosuri et al, 2014.

Note: This script will also output the statistics used to generate Supp Table 1.

**illuminaPerfSeqAnalysis.m**

A script to analyze constructs that are perfect matches to the designed oligonucleotide library. (Brophy et al. 2015).

**Usage** (MATLAB script; requires MATLAB):

illuminaPerfSeqAnalysis(UNS, B1, B2, B3, B4)

**Required input files:**

UNS <seqfile> Input file of perfect reads in unsorted library. Sequence input xlsx format.

B1 <seqfile> Input file of perfect reads in Bin 1 library. Sequence input xlsx format.

B2 <seqfile> Input file of perfect reads in Bin 2 library. Sequence input xlsx format.

B3 <seqfile> Input file of perfect reads in Bin 3 library. Sequence input xlsx format.

B4 <seqfile> Input file of perfect reads in Bin 4 library. Sequence input xlsx format.

**Output files:**

‘Enrichment.csv’ Tab delimited text file, of enrichment for all sequences in sample by bin. Oligonucleotides in same order as input files. Best viewed in Excel or equivalent. Column headers as follows:

Unsorted sample Enrichment of oligonucleotides in the unsorted library.

Bin 1 Enrichment of oligonucleotides in bin 1.

Bin 2 Enrichment of oligonucleotides in bin 2.

Bin 3 Enrichment of oligonucleotides in bin 3.

Bin 4 Enrichment of oligonucleotides in bin 4.

‘Medians.csv’ Tab delimited text file, of statistics for parts in each bin. Best viewed in Excel or equivalent.

Column headers as follows: Row headers as follow:

Unsorted sample First quartile promoter strength.

Bin 1 Median promoter strength.

Bin 2 Third quartile promoter strength.

Bin 3 First quartile forward terminator strength.

Bin 4 Median forward terminator strength.

Third quartile forward terminator strength.

First quartile reverse terminator strength.

Median reverse terminator strength.

Third quartile reverse terminator strength.

**illuminaSeqCoverageGrid.m**

A script to create heatmaps showing counts of oligonucleotides in each bin and the unsorted library (Brophy et al. 2015).

**Usage** (MATLAB script; requires MATLAB):

illuminaEnrichmentGrid(UNS,B1,B2,B3,B4,Order)

**Required input files:**

UNS <seqfile> Input file of perfect reads in unsorted library. Sequence input xlsx format.

B1 <seqfile> Input file of perfect reads in Bin 1 library. Sequence input xlsx format.

B2 <seqfile> Input file of perfect reads in Bin 2 library. Sequence input xlsx format.

B3 <seqfile> Input file of perfect reads in Bin 3 library. Sequence input xlsx format.

B4 <seqfile> Input file of perfect reads in Bin 4 library. Sequence input xlsx format.

Order <seqfile> Input file of ordered promoters and terminators for the enrichment grid.

**Output:**

Heatmap showing bin in which each oligonucleotide is most enriched.

**TI_boundary_value_solver_FNL.m**

A script to solve the transcriptional interference model (Brophy et al. 2015). Parameters within the model can be changed to simulate different promoters, εs, and distance between promoters *N*.

**Output:**

Results of the model. Rows and columns are different forward and antisense promoter combinations.
